# Image-Guided Hydrodynamic Gene Delivery: Current Status and Future Directions

**DOI:** 10.3390/pharmaceutics7030213

**Published:** 2015-08-21

**Authors:** Kenya Kamimura, Takeshi Yokoo, Hiroyuki Abe, Yuji Kobayashi, Kohei Ogawa, Yoko Shinagawa, Ryosuke Inoue, Shuji Terai

**Affiliations:** Division of Gastroenterology and Hepatology, Graduate School of Medical and Dental Sciences, Niigata University, Niigata 951-8510, Japan; E-Mails: t-yokoo@med.niigata-u.ac.jp (T.K.); hiroyukiabe@med.niigata-u.ac.jp (H.A.); yuji@med.niigata-u.ac.jp (Y.K.); kogawa@med.niigata-u.ac.jp (K.O.); yoko-s@med.niigata-u.ac.jp (Y.S.); r-inoue@med.niigata-u.ac.jp (R.I.); terais@med.niigata-u.ac.jp (S.T.)

**Keywords:** gene therapy, hydrodynamics-based gene delivery, DNA, clinical application

## Abstract

Hydrodynamics-based delivery has been used as an experimental tool to express transgene in small animals. This *in vivo* gene transfer method is useful for functional analysis of genetic elements, therapeutic effect of oligonucleotides, and cancer cells to establish the metastatic cancer animal model for experimental research. Recent progress in the development of image-guided procedure for hydrodynamics-based gene delivery in large animals directly supports the clinical applicability of this technique. This review summarizes the current status and recent progress in the development of hydrodynamics-based gene delivery and discusses the future directions for its clinical application.

## 1. Hydrodynamics-Based Gene Delivery in Small Animals

**Figure 1:**
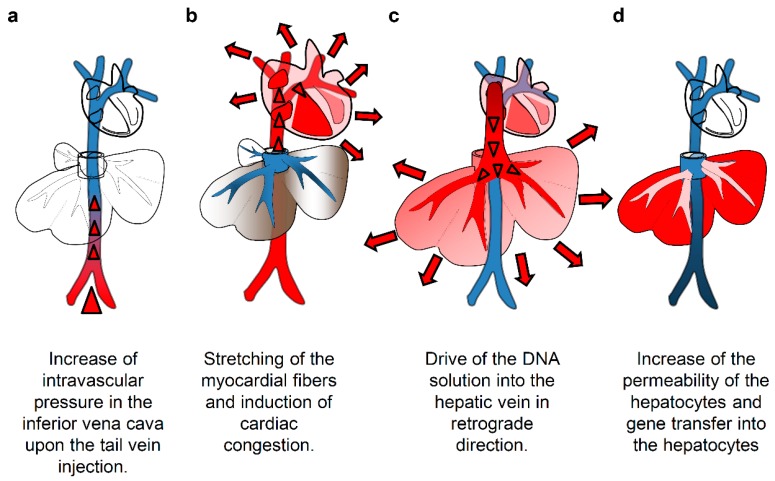
Mechanism of hydrodynamics-based delivery to the mouse liver. (**a**) The intravascular pressure in the inferior vena cava increases upon the tail vein injection of the solution; (**b**) induction of cardiac congestion and accumulation of the solution in the inferior vena cava; (**c**) flow of the injected solution into the liver in retrograde and pushing blood preexistent toward portal side; (**d**) pressure-mediated increase of the membrane permeability and transfer of gene into hepatocytes.

Hydrodynamics-based delivery utilizes hydrodynamic pressure induced by the volume and flow of injection as the driving force to facilitate intracellular gene transfer [1,2]. The original method of hydrodynamic gene delivery (HGD) in small animals involves tail vein injection of plasmid DNA solution in a volume equal to 8%–10% of the body weight (BW) in 5–7 s [1,3]. Among the various organs showing considerably high levels of transgene expression, the liver shows the highest level, and about 40% hepatocytes are transfected by a single injection of less than 50 µg of plasmid DNA [4]. The mechanism of HGD is shown in Figure 1. The injected plasmid solution directly goes to the heart, inducing cardiac congestion and backflow to the inferior vena cava (IVC) and hepatic veins. This solution then reaches the sinusoids; the hydrodynamic pressure expands the liver, enlarges the fenestrae of the endothelium, and forces invagination of the cellular membrane of the hepatocytes to allow the DNA to move into the cytoplasm [5] (Figure 1). In small animals, the opened cellular membrane is smoothly resealed usually within a few minutes, thereby trapping the DNA within the cells. The enlarged liver returns to its original size within 24 h [6]. Hepatic enzymes, including aspartate aminotransferase, alanine aminotransferase, and lactate dehydrogenase, exhibit a transient increase in the serum after the injection and return to normal levels within a few days [6].

Evidence collected thus far shows that HGD has a short-term effect in animals [3]. No long-term effect on animal health has been reported. The significantly higher transgene expression observed in the liver following HGD via the tail vein has been attributed to several features that are unique to the liver, including the presence of fenestrated sinusoids, the absence of a basement membrane, the high capacity of hepatocytes for gene expression, proximity to the IVC, low blood pressure, and the large surface area of hepatocytes facing the lumen. After the procedure, regular blood circulation resumes, the delivered transgene is expressed, and the gene products appear in transfected cells. While the liver shows the highest level of transgene expression in mice, other organs and tissues including kidney, muscles, and even tumors show gene expression, which indicates the applicability of the procedure for various diseases [7,8,9].

## 2. Experimental Applicability of Hydrodynamic Gene Delivery

Because of its simplicity, high efficiency, and reproducibility, hydrodynamics-based delivery has been utilized in scientific research for the delivery of DNA, small interfering RNA (siRNAs), proteins, small compounds, viral vectors, and even cancer cells. Recently, Li *et al.* reported the utility of the procedure for the development of mouse models of multiple metastatic tumors in the liver, kidney, and lung [10]. Hydrodynamics-based delivery of murine melanoma cells (B16-F1), murine breast cancer cells (4T1), and murine renal cell carcinoma (RENCA) resulted in liver, kidney, and lung metastatic tumors, whereas slow injection of these cells resulted in tumors only in the lung [10]. In HGD, the only requirement for gene delivery is a plasmid containing the expression cassette. Persistent gene expression is dependent on the regulatory element in the plasmid, and modifications in this region, such as a combination of albumin promoter and alpha-fetoprotein enhancer introns, can significantly contribute to sustained gene expression [11]. HGD is also useful for delivering large DNA fragments [12] of therapeutic genes as tested in small animal models for various disease models including hepatitis, hematological disorders, metabolic disease, infectious disease, muscle disease, and malignant diseases [4,13,14,15,16,17,18,19,20,21,22,23,24,25,26,27,28,29,30,31,32,33,34,35,36,37,38,39,40,41,42,43,44,45,46]. HGD is also an effective tool in studies of genetic immunization and treatment of viral infection [47,48]. In addition, it has been used in the establishment of small animal disease models for hepatitis, liver fibrosis, liver cancer, sepsis, and lipidemia [49,50,51,52,53,54,55].

Based on the promising results of gene expression studies conducted using small rodents, efforts have been made to apply this method to clinical gene therapy. Establishment of safe and reproducible procedures is an essential step in the project. Key factors that must be addressed include the following: (1) reduction in the injection volume, (2) therapeutic effect, and (3) reproducibility of the procedure that can be performed by any physician. To address these points, we have developed an image-guided, computer-assisted HGD procedure and have shown its safe and effective use for gene delivery in the liver and muscles of pigs and dogs [56,57,58,59].

## 3. Clinical Applicability of Image-Guided Hydrodynamic Gene Delivery

**Figure 2:**
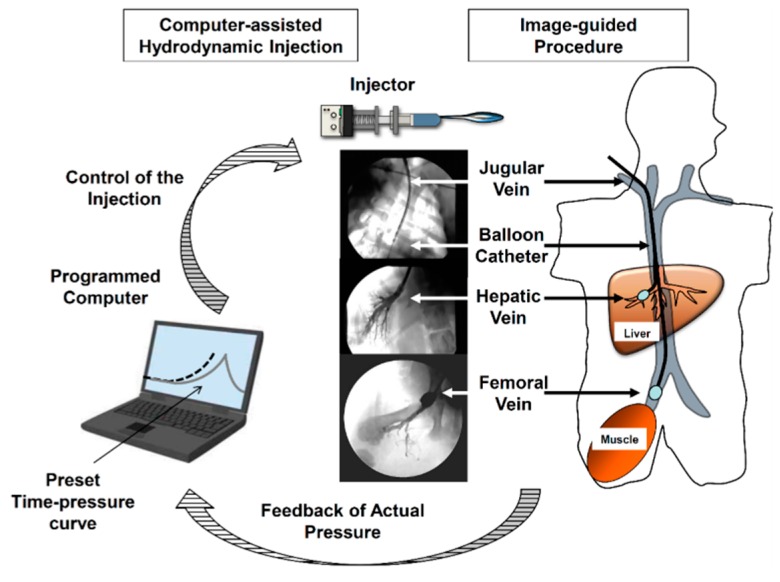
Schematic presentation of computer-assisted and image-guided hydrodynamics-based delivery. Balloon catheter is located at the proper section of hepatic vein (for gene transfer to the liver) or femoral vein (for gene transfer to skeletal muscle). Pressure upon the hydrodynamics-based delivery is transduced from the sensor placed inside of the target vasculature to the computer. Then the computer starts the injection and control the injection speed according to the real-time pressure. The photo images represent the visualization of vascular structure at the insertion site using phase contrast medium (Modified from [64] with permission. Copyright Future Medicine Ltd. 2015).

**Figure 3:**
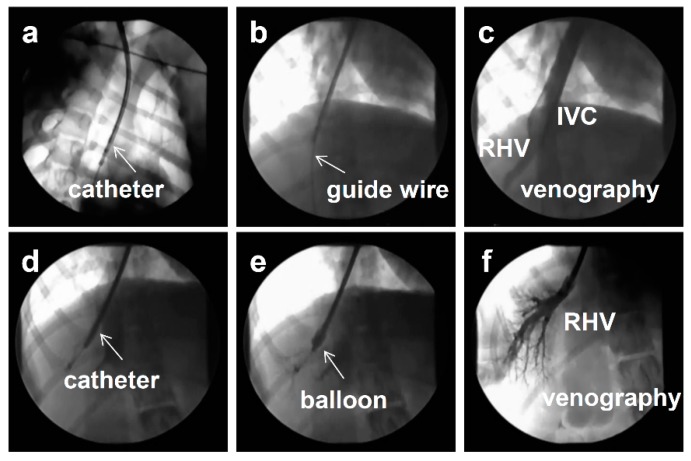
Image-guided procedure for hydrodynamics-based delivery. (**a**) A short sheath is inserted into the jugular vein, followed by an insertion of a balloon catheter. (**b**) Site-specific insertion of guide-wire into the target lobular hepatic vein. (**c**,**d**) Confirmation of target hepatic vein using a small amount of phase contrast. RHV, right hepatic vein, IVC, inferior vena cava. (**e**) Inflation of the balloon on the tip of the catheter to block the backflow. (**f**) Verification of the obstruction of blood flow by injecting a small volume of the phase contrast medium into the vasculature through the catheter.

The image-guided procedure was developed to reduce the injection volume (Figure 2), because an injection volume of approximately 10% of the BW (~5 L for a 50-kg man) is considered impractical and unsafe. Some modifications of the original procedure have been reported. For example, Eastman *et al.* demonstrated that a volume of 15 mL/kg can be safely injected into an isolated liver in rabbits [60]. Similar results were reported by several studies using small pigs as an animal model [61,62,63]. However, due to procedural difficulties, the reproducibility of the procedure and the efficiency of gene delivery remained unclear. To further extend the clinical applicability of the procedure, Kamimura *et al.* performed site-specific gene delivery to the livers [56] and muscles [57] of 20-kg pigs by combining with a clinically well-established X-ray image-guided method of catheter insertion at the site of target organs [56,57]. The procedure involved insertion of a short sheath into the jugular vein, followed by insertion of a balloon catheter (Figure 3a) into the target lobular hepatic vein or femoral vein using a guide wire (Figure 3b–d). The balloon was then inflated by injecting a small amount of the phase contrast medium (Figure 3e). Thereafter, obstruction of blood flow was verified by injecting a small volume of the phase contrast medium into the vasculature through the catheter (Figure 3f), followed by HGD. The injection was given specifically to a localized area in the liver, enabling a significant reduction in the injection volume from 10% of the BW to 1.25% of the BW in pigs [56,58]. Target-specific gene delivery was confirmed at the area near the injection site. The procedure has also been applied to gene delivery in the muscles of pigs. Gene delivery parameters, including injection site, flow rate, and volume, have been optimized and the clinical applicability of the procedure in terms of safety and efficiency has been shown in large pigs [58] and dogs [59]. Using optimized conditions, human alpha-1 antitrypsin gene expression was achieved in pig liver for more than two months after gene delivery [58]. Physiological parameters, including heart rate, blood pressure, oxygen saturation, and body temperature, remained normal during and after hydrodynamic injection in these animal studies [59]. In addition, no changes in BW were observed in dogs during the experimental period, which included three hydrodynamic injections over 6 weeks at 2-week time intervals. Following liver-targeted HGD, PCR was used to assess systemic distribution of the plasmid DNA; results showed no gene transfer to cells beyond the target organ [58,59]. In addition, no allergic symptom or change in the animal condition was observed following repeated HGD using the same genes [58,59]. These results support the clinical applicability of the image-guided HGD procedure in terms of its safety and efficiency for gene delivery.

## 4. Clinically Applicable Reproducible Procedure

To achieve clinical reproducibility in terms of the therapeutic effect, development of an automatic computer-controlled injection device is essential. Previous studies have identified the injection time–intravascular pressure curve as the key parameter for HGD [6]. Suda *et al.* developed a first-generation computer-controlled injection device using CO_2_ gas as a physical force to control the time–pressure curve of HGD [65]. The intravascular pressure in the animal body upon injection is transmitted to the computer through the pressure detector placed at the tip of the injection catheter. The computer turns the sustained injection CO_2_ pressure on or off according to the detected intravascular pressure to achieve the desired level of intravascular pressure or to stop the injection once the level is achieved [65]. The injector has shown reproducible gene delivery to muscle and liver in pigs [56,57]. Kamimura *et al.* have recently developed a new electric motor-driven injector [59,66]. The injector self-adjusts the injection power depending on the real-time feedback of the actual pressure in the body and reproduces the preloaded time–pressure curves installed in the computer prior to the injection. The major advantage of using this injector is that it eliminates the risk of CO_2_ embolism or unexpected high intravascular pressure that can occur due to the sustained injection CO_2_ pressure and damage the injection-targeted organs [59,66]. Recently, it has been tested in large animals and shown to be safe, reproducible, and efficient for gene delivery in dogs [59]. Combination of an image-guided procedure and a computer-controlled injection device will allow physicians to apply this procedure in the clinic (Figure 2).

## 5. Conclusions and Future Perspectives

Significant progress has been made in a variety of gene delivery systems to date. While viral vectors are highly effective and have been used in approximately 67% of clinical trials, the possibility of carcinogenesis and development of an immune response remain the largest hurdles to clinical application [64,67,68,69]. For nonviral vector-based gene delivery used in 24% of clinical trials, a variety of compounds have been used; however, their effectiveness in gene delivery remains significantly lower than that of viral vectors. Gene delivery methods using a physical approach, including needle injection, ballistic DNA injection, electroporation, sonoporation, photoporation, magnetofection, and HGD, are a part of the nonviral vector-based gene delivery approach; these are relatively new and have demonstrated their potential to transfer DNAs directly into the cells in the target organs.

Among these methods, HGD has been extensively studied in small animals. The simplicity and effectiveness of the approach has encouraged scientists and physicians to further develop hydrodynamics-based gene therapy. The recent progress in image-guided catheterization-based procedures has enabled physicians to perform HGD to specific target organs. This site-specific gene delivery with reduced injection volume and the development of computer-controlled injection devices enables safe, reproducible, and efficient gene delivery. Recently, a study using liver-targeted computer-controlled HGD showed no change in systemic inflammatory cytokines after injection, whereas an increase in myokines was observed due to the stretching of the vascular structure upon injection [59]. These results support the clinical applicability of HGD, and recently the more clinically applicable computer-controlled injector has been developed. The pre-clinical trial in the large animals using the new injector is ongoing (unpublished data). Further efforts must be made to develop the procedure in non-human primates as a human model and to establish plasmids that are clinically suitable for long-term gene expression.
